# Simulation of Legionnaires’ disease prospective spatiotemporal cluster detection, Allegheny County, Pennsylvania, USA

**DOI:** 10.1017/S0950268818002789

**Published:** 2018-10-18

**Authors:** L. T. Orkis, E. R. Peterson, M. M. Brooks, K. J. Mertz, L. H. Harrison, J. E. Stout, S. K. Greene

**Affiliations:** 1 Department of Epidemiology, University of Pittsburgh Graduate School of Public Health, Pittsburgh, Pennsylvania, USA; 2 Allegheny County Health Department, Bureau of Assessment, Statistics, and Epidemiology, Pittsburgh, Pennsylvania, USA; 3 New York City Department of Health and Mental Hygiene, Bureau of Communicable Disease, Queens, New York, USA; 4 Department of Epidemiology, Infectious Diseases Epidemiology Research Unit, University of Pittsburgh Division of Infectious Diseases, Pittsburgh, Pennsylvania, USA; 5 Special Pathogens Laboratory, Pittsburgh, Pennsylvania, USA; 6 Department of Civil and Environmental Engineering, University of Pittsburgh Swanson School of Engineering, Pittsburgh, Pennsylvania, USA

**Keywords:** Epidemiology, Legionaire's disease, surveillance

## Abstract

Legionnaires’ disease (LD) incidence in the USA has quadrupled since 2000. Health departments must detect LD outbreaks quickly to identify and remediate sources. We tested the performance of a system to prospectively detect simulated LD outbreaks in Allegheny County, Pennsylvania, USA. We generated three simulated LD outbreaks based on published outbreaks. After verifying no significant clusters existed in surveillance data during 2014–2016, we embedded simulated outbreak-associated cases into 2016, assigning simulated residences and report dates. We mimicked daily analyses in 2016 using the prospective space-time permutation scan statistic to detect clusters of ⩽30 and ⩽180 days using 365-day and 730-day baseline periods, respectively. We used recurrence interval (RI) thresholds of ⩾20, ⩾100 and ⩾365 days to define significant signals. We calculated sensitivity, specificity and positive and negative predictive values for daily analyses, separately for each embedded outbreak. Two large, simulated cooling tower-associated outbreaks were detected. As the RI threshold was increased, sensitivity and negative predictive value decreased, while positive predictive value and specificity increased. A small, simulated potable water-associated outbreak was not detected. Use of a RI threshold of ⩾100 days minimised time-to-detection while maximizing positive predictive value. Health departments should consider using this system to detect community-acquired LD outbreaks.

## Introduction

Legionnaires’ disease (LD) is pneumonia caused by *Legionella* species that disproportionately affects elderly and immunocompromised persons and can be fatal [1]. Transmission occurs primarily through inhalation of aerosolised droplets from a contaminated water source. Known sources include large building water systems, cooling towers, soil, hot tubs and residential potable water systems [1]. LD outbreaks can result in substantial morbidity and mortality [2].

Rapid detection of disease clusters is critical to prevention and control. Statistical cluster detection has been shown to successfully identify LD outbreaks quickly and accurately using both prospective and retrospective methods [3–5]. The New York City Department of Health and Mental Hygiene (NYC DOHMH) has performed daily analyses since 2014 using the prospective space-time permutation scan statistic available through a free software program called SaTScan to detect clusters in 35 reportable communicable diseases, including legionellosis [6]. Advantages of using this scan statistic method include that it does not impose any artificial boundaries on the spatial or temporal extent of a cluster, does not require population-at-risk data and accounts for multiple testing [7]. This scan statistic method has also been applied effectively for surveillance for clusters of dead birds as an early warning of West Nile virus activity, hospital emergency department visits, ambulance dispatch calls, pharmacy sales, shigellosis and campylobacteriosis [7–13].

Several outbreaks were first detected using the NYC DOHMH system, including in 2015 the second largest community-acquired LD outbreak in the USA, which was associated with 138 cases and 16 deaths [6, 14]. In this instance, the NYC DOHMH system automatically detected a significant cluster 3 days before NYC DOHMH staff independently noted an increase in LD cases and 4 days before hospital staff notified NYC DOHMH of an increase in LD among emergency department patients [6, 14]. A resource-intensive epidemiologic, environmental and laboratory investigation identified the source, a cooling tower on a hotel [14]. The NYC DOHMH cluster detection system contributed to the timeliness of outbreak detection, investigation and mitigation and was useful for tracking the scope of the outbreak after initial detection, as additional cases were reported.

In the USA, only 4% of LD cases have been shown to be outbreak-associated [15]. Allegheny County, Pennsylvania, USA, with a population of 1.2 million people including the city of Pittsburgh and its surrounding suburbs [16], had an age-adjusted legionellosis rate of 4.4 per 100 000 in 2009 [17], which was fourfold higher than the national legionellosis rate [2]. About 25% of cases diagnosed during 2013–2016 among Allegheny County residents were healthcare-associated; the remaining 75% of cases were classified as community-acquired sporadic (non-outbreak-associated). Allegheny County Health Department (ACHD) staff routinely conduct patient interviews to identify risk factors and then review collected data to assess common exposures among cases. However, detecting community-acquired clusters through human review of case investigation data relies on astute staff noticing unusual clustering in time and space and recognising links between cases, is time- and resource-intensive to conduct and can lag days to weeks behind an increase in reported cases. This issue can be exacerbated in densely populated urban areas with large numbers of background cases.

During 2003–2017, only one community-acquired LD outbreak, occurring in 2008, was identified in Allegheny County. Traditional patient interview-based surveillance methods have not identified common exposures among other community-acquired Allegheny County LD cases. Relying solely on human review and descriptive epidemiology to detect clusters could result in missing clusters, such that cases are assumed to be sporadic rather than investigated as potentially having a common source. More timely detection of LD cases clustered in space and time that might signify a cooling tower-associated outbreak would lead to faster outbreak investigation, source mitigation and disease prevention. The objective of this study was to determine the adaptability, utility and performance of NYC DOHMH's prospective cluster detection system using its particular SaTScan parameter settings to detect LD outbreaks of various sizes in Allegheny County, a smaller, less densely populated jurisdiction.

## Methods

Data on legionellosis cases among Allegheny County residents reported by laboratories and healthcare providers during 2014–2016 were obtained through Pennsylvania's National Electronic Disease Surveillance System (PA-NEDSS). Legionellosis comprises two conditions caused by *Legionella*: LD and Pontiac fever, which is a milder febrile illness and therefore, less commonly diagnosed and reported. A confirmed case of legionellosis is defined by the Council of State and Territorial Epidemiologists as a clinically compatible illness confirmed by laboratory culture, urine antigen or antibody seroconversion [18]. Age-adjusted annual incidence was calculated using direct standardisation and weighting to the US 2000 standard population [19]. Date of report and latitude and longitude coordinates of the centroid of the census tract of residence were used to represent the temporal and spatial aspects of each case.

Three simulated outbreaks were created based on data published on community-acquired LD outbreak investigations [20–22]. These outbreaks were selected because they represent three distinct LD community-acquired outbreak types that could potentially be detected by the space-time permutation scan statistic: (1) 50 cases presumed to be associated with a cooling tower occurring rapidly over 38 days, (2) 84 cooling tower-associated cases occurring at a moderate speed over 82 days and (3) 10 community potable water system-associated cases occurring slowly over 163 days. The outbreaks varied by environmental source, number of cases, duration, the growth of epidemic curve, the radius of the affected area and season (Table 1). The cases from each individual outbreak were inserted into Allegheny County baseline data based on a simulated report date to mimic the published epidemic curve. No published epidemic curve for simulated outbreak 3 was available [22] and, therefore, we used information on the timing of cases available in the publication to simulate an epidemic curve.

**Table 1.** Legionnaires’ disease simulated outbreak characteristics based on published outbreak investigations

Outbreak simulation	Outbreak location	Population size	Case count	Duration	Speed of growth	Maximum temporal cluster size	Environmental source	Radius encompassing all outbreak case residences	Season
# 1 [20]	Edinburgh, scotland	495 360	50	38 days	Rapid	30 days	Cooling tower	6 miles	Early summer
# 2 [21]	Pas-de-Calais, France	1 452 590	84	82 days	Moderate	30 days, 180 days	Cooling tower	7.5 miles	Fall
# 3 [22]	New Jersey, exact location not disclosed	Not described	10	163 days	Slow	180 days	Potable water	1 mile	Summer

Each published outbreak used for a simulation included a point map of the spatial distribution of case residences. For each simulated outbreak, we mimicked the published outbreak spatial distribution by calculating the distribution of published outbreak cases within circular bands of increasing radius centred on the outbreak source (i.e. ‘outbreak radii’) and then assigning locations to simulated outbreak cases to achieve a similar distribution relative to the simulated outbreak area. The publication used as the basis for simulated outbreak 2 also included the spatial distribution of cases during two time periods. We used this information to further refine the case spatial distribution of simulated outbreak 2.

We analysed the simulated study data for Allegheny County, which included baseline or routine public health surveillance data spiked with simulated outbreak cases, using a SAS program created by NYC DOHMH [6] and modified for use by ACHD. The original NYC DOHMH SAS program was easily modified by an ACHD epidemiologist with intermediate SAS skills. Minor modifications included editing portions of the original code to conform to PA-NEDSS-specific nuances, removing code related to secondary addresses and editing code referencing NYC-specific geographic resolutions [6].

The space-time permutation scan statistic evaluates potential clusters as space-time cylinders, with the circular base representing space and the height representing time, encompassing all possible clusters within the spatial and temporal limits defined in the analysis parameter settings. A likelihood ratio-based test statistic is calculated for each cylinder as a function of observed and expected case counts, inside and outside the cylinder. The cluster with the maximum test statistic is identified. A large number of random replications of the input dataset are generated under the null hypothesis that the same process generated disease counts inside and outside the cylinder [7]. The *P*-value for the cluster with the maximum test statistic is obtained through Monte Carlo hypothesis testing, by comparing the rank of the maximum likelihood from the real case dataset with the maximum likelihoods from the randomly generated datasets. A recurrence interval (RI) is calculated as the reciprocal of the *P*-value [23]. If analyses are conducted daily, the RI represents the number of days of analyses required for the expected number of clusters at least as unlikely as the observed cluster to be equal to 1 by chance alone [24].

The NYC DOHMH standard maximum spatial cluster size of 50% of all cases reported during the study period was used for this analysis. The SaTScan method also requires selection of a maximum temporal cluster size, which should be short (e.g. 30 days) to have optimal power to detect a rapidly growing outbreak like simulated outbreak 1, or long (e.g. 180 days) to be able to detect a slowly growing outbreak like simulated outbreak 3 [6]. The epidemic curve for simulated outbreak 2 was neither clearly rapidly nor slowly growing, so we did not know a priori which maximum temporal window would be preferable. Therefore, we analysed simulated outbreak 2 using both 30- and 180-day maximum temporal windows to determine which performed better. For maximum temporal cluster sizes of 30 and 180 days, we used 365 and 730 days of historical baseline data, respectively.

We assessed 2014–2016 Allegheny County legionellosis case reports for previously unidentified clusters through a retrospective analysis using the space-time permutation scan statistic in SaTScan. These years were analysed given they represented the full range of possible baseline data used for prospective analyses (e.g. when analysing a period ending on 1 January 2016 using a maximum temporal cluster size of 180 days, the case file for analysis would include cases for a 730-day period starting 2 January 2014). We spiked the 2016 baseline data with cases for each of the three simulated LD outbreaks. Analyses for each of the three outbreaks were performed separately. We mimicked daily prospective analyses for the entire year of 2016 and defined signals using three RI thresholds: 20 days (a low threshold that is sometimes nevertheless used as it corresponds to *P* < 0.05, failing to account for repeated analyses performed daily instead of once), 100 days (the threshold used by NYC DOHMH for reportable diseases [6]) and 365 days (a threshold sometimes used for less specific data streams, e.g. syndromic surveillance data [25, 26]). Analysis days were restricted to days in which a baseline or simulated case was reported, as only these days had the potential to yield informative signals. Analysis days with at least one cluster exceeding the RI threshold were classified as positive, while analysis days with no clusters exceeding the RI threshold were classified as negative. A true positive required ⩾3 simulated cases included in the cluster detected. A false positive required <3 simulated cases included in the cluster detected. A true negative required <3 simulated cases reported in the maximum temporal window OR zero simulated cases reported that day. A false negative required ⩾3 simulated cases reported in maximum temporal window AND ⩾1 simulated case reported that day.

These daily analysis classifications were used to calculate the validity statistics of sensitivity, specificity, positive predictive value (PPV) and negative predictive value (NPV) at the three RI signalling thresholds for each simulated outbreak. Sensitivity was defined as the proportion of true positive daily analyses amongst all daily analyses that met cluster signalling criteria. Specificity was defined as the proportion of true negative daily analyses amongst all daily analyses that did not meet cluster signalling criteria. PPV was defined as the proportion of true positive daily analyses amongst all signalled daily analyses. NPV was defined as the proportion of the true negative daily analyses amongst all non-signaled daily analyses. Time to outbreak detection was calculated for each simulated outbreak by subtracting the earliest outbreak detection date from the report date of the third simulated outbreak-associated case, assuming a common source, community-acquired outbreak reasonably could not be recognised nor a source identified with fewer than three reported cases. All analyses were performed using SAS (v.9.4) and SaTScan (v.9.4.4).

## Results

**Fig. 1.**
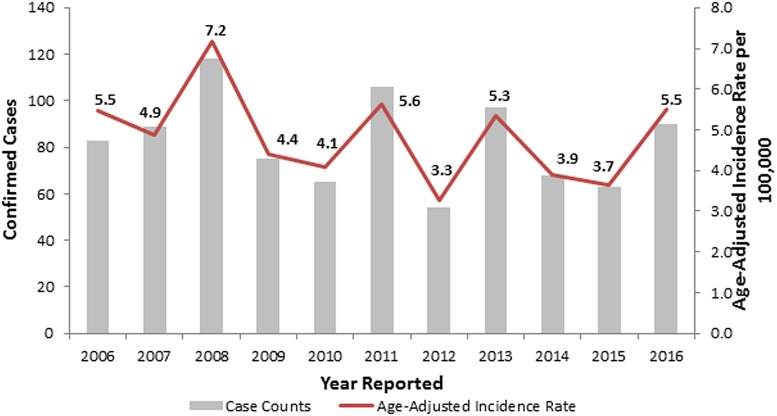
Confirmed legionellosis cases and age-adjusted legionellosis incidence rates, Allegheny County, Pennsylvania, USA, 2006–2016.

During 2006–2016, the observed number of legionellosis cases reported per year in Allegheny County ranged between 54 and 118, and 90 cases were reported in 2016 (Fig. 1). When retrospectively analyzing these 2014–2016 Allegheny County legionellosis surveillance data, no clusters were detected.

**Fig. 2.**
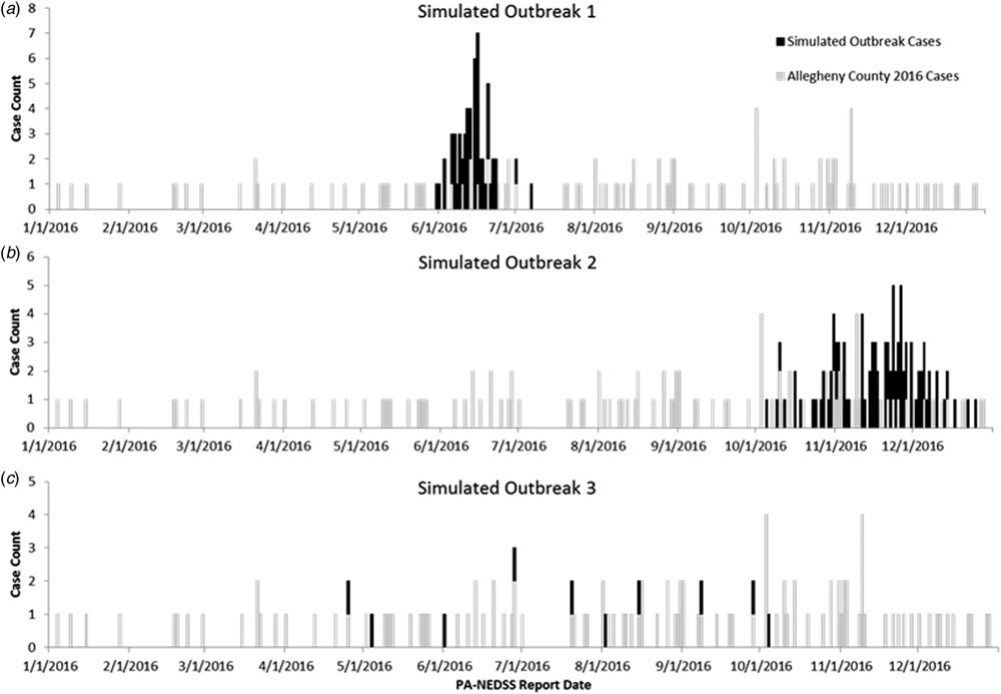
(a–c) Observed baseline and simulated outbreak-associated legionellosis cases, Allegheny County, Pennsylvania, USA, 2016.

**Fig. 3.**
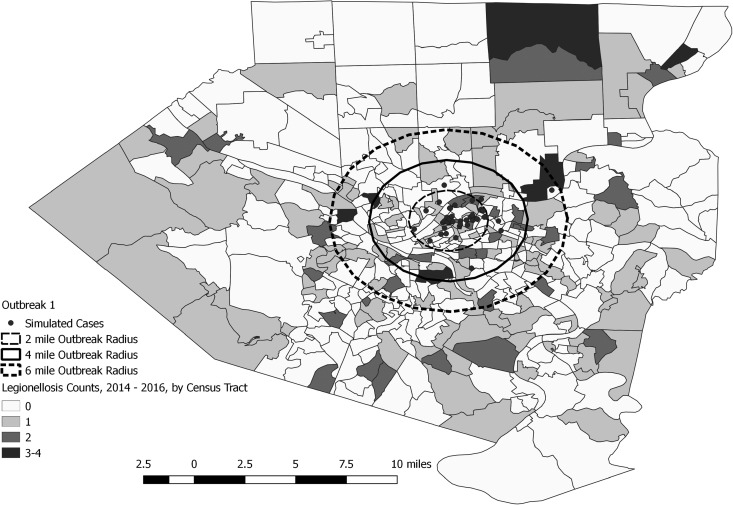
Simulated outbreak 1-associated Legionnaires’ disease cases, simulated outbreak radii and legionellosis cases by census tract, 2014–2016, Allegheny County, Pennsylvania, USA.

**Fig. 4.**
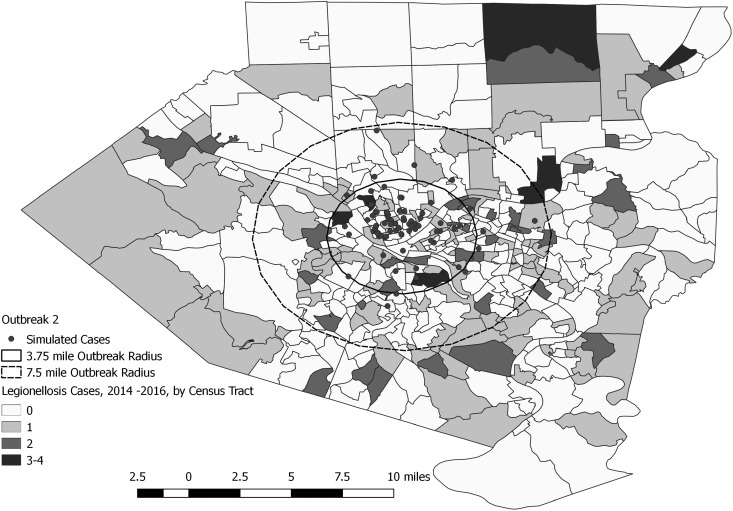
Simulated outbreak 2-associated Legionnaires’ disease cases, simulated outbreak radii and legionellosis cases by census tract, 2014–2016, Allegheny County, Pennsylvania, USA.

**Fig. 5.**
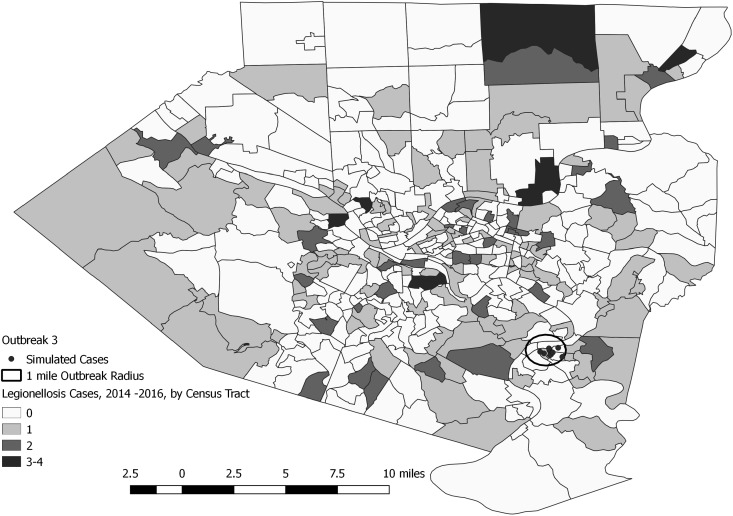
Simulated outbreak 3-associated Legionnaires’ disease cases, simulated outbreak radii and legionellosis cases by census tract, 2014–2016, Allegheny County, Pennsylvania, USA.

A total of 144 outbreak cases were added as part of three separate outbreak simulations (Figs. 2–5). The time-to-detection using a RI ⩾ 100-day threshold was 5 days for outbreak 1 and 38 days using a 30-day maximum temporal window for outbreak 2; outbreak 3 was not detected (Table 2). Time-to-detection was shortened by using a lower RI threshold. Using a RI ⩾ 100-day threshold, time-to-detection was shortened to 33 days for outbreak 2 when a 180-day maximum temporal window and longer baseline period were used (Table 2). The maximum recurrence interval observed for outbreak 1 was over 3000 years using a 30-day maximum temporal window (Table 3). The maximum recurrence interval observed for outbreak 2 was higher when using a 180-day compared with a 30-day maximum temporal window (Table 3).

**Table 2.** Time-to-detection, defined as days from third outbreak-associated case report to first signal exceeding recurrence interval (RI) threshold, for three simulated Legionnaires’ disease outbreaks, Allegheny County, 2016

	30-day max temporal window			180-day max temporal window		
	RI ⩾ 20	RI ⩾ 100	RI ⩾ 365	RI ⩾ 20	RI ⩾ 100	RI ⩾ 365
Outbreak simulation 1	1 day	5 days	5 days	n/a^a^	n/a^a^	n/a^a^
Outbreak simulation 2	22 days	38 days	Not detected	33 days	33 days	36 days
Outbreak simulation 3	n/a^a^	n/a^a^	n/a^a^	Not detected	Not detected	Not detected

a A maximum temporal window was chosen for each simulated outbreak based on the temporal span of the outbreak. The n/a designation was listed when a maximum temporal window was not used for analysis of a particular outbreak. Outbreak 2 did not clearly fit an appropriate maximum temporal window, thus both 30-day and 180-day were used.

**Table 3.** Maximum recurrence interval (days) observed for a cluster of greater than three simulated cases

	30-day max temporal window	180-day max temporal window
Outbreak simulation 1	1 233 688	n/a
Outbreak simulation 2	345	5433
Outbreak simulation 3	n/a	Not detected

Signals identified spatial and temporal extents of clusters of excess disease activity but did not attribute individual cases within clusters as outbreak-related *vs.* background. The maximum number of background cases included in any identified cluster was two, with total numbers of observed cases (i.e. simulated outbreak cases plus background cases within such clusters) ranging from 17 to 38 cases.

Using a 30-day maximum temporal window and an RI signaling threshold ⩾20 days or ⩾100 days, all validity statistics for simulated outbreak 1 daily analyses were ⩾90%; thus, few false negative and false positive days were produced (Table 4). Using a 30-day maximum temporal window, the sensitivity of outbreak 2 detection was low using a RI ⩾ 20 days threshold and very low using a RI ⩾ 100 day threshold, whereas all other validity statistics were ⩾64% (Table 5). When using a 180-day maximum temporal window and any RI signalling threshold, the sensitivity of outbreak 2 daily analyses was ⩾43%. The other validity statistics were ⩾76% (Table 5). Using a 180-day maximum temporal window and any RI signalling threshold, outbreak 3 was not detected, so sensitivity was 0%. The specificity was ⩾97% (Table 6).

**Table 4.** Simulated outbreak 1 daily analyses validity statistics (*n* = 105 days with any reported cases in 2016)

	30-day max temporal window		
	RI ⩾ 20 (%)	RI ⩾ 100 (%)	RI ⩾ 365 (%)
Sensitivity	100	95.2	90.4
Specificity	98.8	100	100
Positive predictive value	95.4	100	100
Negative predictive value	100	98.8	97.7

**Table 5.** Simulated outbreak 2 daily analyses validity statistics (*n* = 125 days with any reported cases in 2016)

	30-day max temporal window			180-day max temporal window		
	RI ⩾ 20 (%)	RI ⩾ 100 (%)	RI ⩾ 365 (%)	RI ⩾ 20 (%)	RI ⩾ 100 (%)	RI ⩾ 365 (%)
Sensitivity	29.5	6.8	0	50.0	50.0	43.2
Specificity	98.8	100	100	98.8	98.8	98.8
Positive predictive value	92.9	100	Undefined	95.7	95.7	95.0
Negative predictive value	72.1	66.4	64.8	78.5	78.5	76.2

**Table 6.** Simulated outbreak 3 daily analyses validity statistics (*n* = 94 days with any reported cases in 2016)

	180-day max temporal window		
	RI ⩾ 20 (%)	RI ⩾ 100 (%)	RI ⩾ 365 (%)
Sensitivity	0	0	0
Specificity	97.7	100	100
Positive predictive value	0	Undefined	Undefined
Negative predictive value	91.3	91.5	91.5

## Discussion

We demonstrated that the space-time permutation scan statistic in SaTScan as applied in the NYC DOHMH cluster detection system identified two larger simulated outbreaks of varying intensity and duration and failed to detect one smaller, slowly growing simulated outbreak in Allegheny County. Outbreak 1 was detected within a few days and outbreak 2, which was slower growing, was detected within a few weeks. The validity statistics of the second simulation were higher when using the 180-day maximum temporal window and 2-year baseline period compared with the 30-day maximum temporal window and 1-year baseline. This observation supports using both the 30-day and 180-day maximum temporal windows for prospective cluster detection since it is unknown in advance whether any given outbreak will be rapidly or slowly growing, although this method does not account for the multiple testing of the same temporal windows up to 30 days in each analysis. Both simulated outbreaks 1 and 2 were based on cooling tower-associated outbreaks, which can be difficult to quickly detect through human review alone.

Simulated outbreak 3 was not detected, as it occurred over a longer period and included few excess cases. Illustrating the type of outbreak, the system is not optimised to detect and highlight the continued importance of astute public health investigators to complement automated detection methods. Through traditional human review of case data, the New Jersey Department of Health detected the outbreak that simulated outbreak 3 after cases occurred in nearby senior apartment buildings. In addition, non-statistical analyses can identify multiple cases within a defined period sharing a common potable water source or building water system; for example, NYC DOHMH runs automated analyses to detect ⩾2 Legionnaires’ disease patients with the same address within a year [27].

Health departments should consider using the prospective space-time permutation scan statistic in SaTScan for routine reportable disease cluster detection, as, in particular, this might help to detect cooling tower-associated LD outbreaks. In practice, the signalling threshold of RI ⩾ 100 days balanced optimising sensitivity (which favours a low RI threshold) and PPV (which favours a high RI threshold). Each outbreak was detected more quickly using an RI threshold of 20 days; however, with the lower PPV associated with this threshold, more false positives were produced that could overextend limited public health resources. This method could also corroborate spatiotemporal trends observed by public health investigators and provide additional evidence to support the need for further investigation. Detecting a significant cluster, using this method should prompt an investigation of a potential source including enhanced patient interviews and environmental sampling. Cooling tower registries, such as the one established in NYC in 2015, can be useful for identifying cooling towers for sampling, especially in dense urban environments where cooling towers are not necessarily visible from the street [28, 29]. Given the circular shape of the scanning window, the space-time permutation scan statistic most successfully detects circular and highly focal outbreaks, such as some LD cooling tower-associated outbreaks. Nevertheless, non-circular shaped outbreaks have been successfully detected by this method [12].

Our assessment has several limitations. First, the PA-NEDSS case report date was the only date used in our simulations mimicking published epidemic curves. Using onset or diagnosis date would have been a better proxy of when the exposure occurred but would have required strong assumptions regarding reporting lags. In practice, report dates can be delayed because of batched electronic laboratory reporting and might have a temporal pattern appreciably different from the date of exposure, which could impact the results of outbreak detection analyses.

Second, the residential address was the only address simulated for each case. NYC DOHMH also performs LD cluster detection using all available address information, including work addresses, which improves sensitivity for detecting clusters where a patient's exposure occurred in a location other than the home [30]. PA-NEDSS does not systematically include data on work address; thus, this analysis reflects a current limitation of PA-NEDSS. However, the home address is often a good approximation of where individuals might have been exposed to *Legionella* and each publication used to simulate outbreaks described case spatial distributions based solely on residential address.

Third, daily analyses were simulated only over 1 year. Results might vary if repeated over several years, given fluctuation in baseline Allegheny County legionellosis case counts, or if repeated by inserting simulated outbreak cases into different parts of Allegheny County with different baseline counts. Only three simulated outbreaks were generated for this analysis, selected to represent three distinct types of community-acquired LD outbreaks. Many simulations of one outbreak type could have been generated with parameter specifications; however, we chose to simulate one outbreak of each type as accurately as possible based on published information including spatial distributions and epidemic curves. Information about the epidemic curve of outbreak 3 was limited and whether our simulation accurately reproduced the outbreak is unknown.

The method used to simulate the spatial distribution of each outbreak has limitations. The outbreaks used for simulation occurred outside of Allegheny County in jurisdictions that differ from Allegheny County in many ways. Creation of these simulations required making assumptions about the spatial distribution of cases that in actuality might take a different form because of differences in Allegheny County population density and distribution, differences in terrain and wind patterns. Also, we did not take into account area-based poverty when considering the spatial distribution of simulated cases. Increased legionellosis rates have been shown to be associated with increased area-based poverty [31–33]. This might have affected our ability to detect increases in case counts relative to baseline legionellosis. If our simulated case clusters were embedded in areas of high poverty, these clusters might have been more difficult to detect given potentially higher baseline legionellosis burden in these areas.

Finally, these findings might not be fully generalisable to jurisdictions with low legionellosis incidence. A small, slowly growing potable water outbreak was not detected in our simulations, but might be easier to detect in other jurisdictions with lower baseline case counts. Similarly, inserting the same simulated cooling tower-associated outbreaks into data from jurisdictions with a lower count of background sporadic cases might result in shorter time-to-detection, higher RIs and improved validity statistics.

## Conclusion

This cluster detection system was easily adapted for use in Allegheny County and quickly detected simulated cooling tower-associated outbreaks that otherwise might have required more time to detect by surveillance methods that rely on the human review of descriptive epidemiology. Health departments should consider adopting this system for improved community-acquired LD outbreak detection and potential disease prevention. In September 2017, Allegheny County began using this system for weekly prospective LD cluster detection.

## References

[1] Cunha BA, Burillo A and Bouza E (2016) Legionnaires’ disease. Lancet 387, 376–385. 26231463 10.1016/S0140-6736(15)60078-2

[2] Garrison LE (2016) Vital signs: deficiencies in environmental control identified in outbreaks of Legionnaires’ disease – North America, 2000–2014. Morbidity and Mortality Weekly Report 65, 576–584. 27281485 10.15585/mmwr.mm6522e1

[3] Rudbeck M (2010) Geographical variation of sporadic Legionnaires’ disease analysed in a grid model. Epidemiology and Infection 138, 9–14. 19527549 10.1017/S0950268809990185

[4] van den Wijngaard CC (2010) Syndromic surveillance for local outbreaks of lower-respiratory infections: would it work? PLoS ONE 5, e10406. 20454449 10.1371/journal.pone.0010406 PMC2861591

[5] Sansom P (2013) A case-association cluster detection and visualisation tool with an application to Legionnaires’ disease. Statistics in Medicine 32, 3522–3538. 23483594 10.1002/sim.5765 PMC3842591

[6] Greene SK (2016) Daily reportable disease spatiotemporal cluster detection, New York City, New York, USA, 2014–2015. Emerging Infectious Disease 22, 1808–1812. 10.3201/eid2210.160097 PMC5038417 27648777

[7] Kulldorff M (2005) A space-time permutation scan statistic for disease outbreak detection. PLoS Medicine 2, e59. 15719066 10.1371/journal.pmed.0020059 PMC548793

[8] Das DMF (2004) Monitoring over-the-counter pharmacy sales for early outbreak detection in New York City. Morbidity and Mortality Weekly Report 53.

[9] Heffernan R (2004) Syndromic surveillance in public health practice, New York City. Emerging Infectious Disease 10, 858–864. 10.3201/eid1005.030646 15200820

[10] Hughes GJ and Gorton R (2013) An evaluation of SaTScan for the prospective detection of space-time Campylobacter clusters in the North East of England. Epidemiology and Infection 141, 2354–2364. 23347688 10.1017/S0950268812003135 PMC9151409

[11] Jones RC (2006) Use of a prospective space-time scan statistic to prioritize shigellosis case investigations in an urban jurisdiction. Public Health Reports 121, 133–139. 10.1177/003335490612100206 PMC1525257 16528945

[12] Kulldorff M (2004) Benchmark data and power calculations for evaluating disease outbreak detection methods. MMWR Supplement 53, 144–151. 15714644

[13] Mostashari F (2003) Dead bird clusters as an early warning system for West Nile virus activity. Emerging Infectious Disease 9, 641–646. 10.3201/eid0906.020794 PMC3000152 12781002

[14] Weiss D (2017) A large community outbreak of Legionnaires’ disease associated With a cooling tower in New York City, 2015. Public Health Reports 132, 241–250. 28141970 10.1177/0033354916689620 PMC5349490

[15] United States Department of Health and Human Services Centers for Disease Control Prevention (2011) Legionellosis – United States, 2000–2009. Morbidity and Mortality Weekly Report 60, 1083–1086. 21849965

[16] United States Census Bureau (2017) Allegheny County, Pennsylvania. [cited 2017 11/10/2017]; Available at https://factfinder.census.gov/faces/nav/jsf/pages/community_facts.xhtml?src=bkmk.

[17] Allegheny County Health Department (2014) *Allegheny County summary of reportable disease 2004–2013*.

[18] United States Department of Health and Human Services Centers for Disease Control and Prevention (2005) *Legionellosis/Legionnaires’ Disease or Pontiac Fever (Legionella pneumophila 2005 Case Definition* October 20, 2017]; Available at https://wwwn.cdc.gov/nndss/conditions/legionellosis/case-definition/2005/.

[19] Klein RJ and Schoenborn CA Age adjustment using the 2000 projected U.S. population. Healthy People 2010 Statistics Notes. 20011–10. 11676466

[20] McCormick D (2012) Public health response to an outbreak of Legionnaires’ disease in Edinburgh, United Kingdom, June 2012. Eurosurveillance 17. 10.2807/ese.17.28.20216-en 22835439

[21] Nguyen TM (2006) A community-wide outbreak of legionnaires disease linked to industrial cooling towers – how far can contaminated aerosols spread? Journal of Infectious Disease 193, 102–111. 10.1086/498575 16323138

[22] Cohn PD (2015) Community outbreak of legionellosis and an environmental investigation into a community water system. Epidemiology and Infection 143, 1322–1331. 25083716 10.1017/S0950268814001964 PMC9507180

[23] Kulldorff M (2018) *SaTScan User Guide* 2018 January 2018 [cited 2018 March 9, 2018]; Available at https://www.satscan.org/cgi-bin/satscan/register.pl/SaTScan_Users_Guide.pdf?todo=process_userguide_download.

[24] Kleinman K (2004) A generalized linear mixed models approach for detecting incident clusters of disease in small areas, with an application to biological terrorism. American Journal of Epidemiology 159, 217–224. 14742279 10.1093/aje/kwh029

[25] Wahnich A (2018) Surveillance for Mosquitoborne Transmission of Zika Virus, New York City, NY, USA, 2016. Emerging Infectious Diseases 24, 827–834. 29664375 10.3201/eid2405.170764 PMC5938798

[26] Kulldorff M and Kleinman K (2015) Comments on ‘a critical look at prospective surveillance using a scan statistic’ by T. Correa, M. Costa, and R. Assuncao. Statistics in Medicine 34, 1094–1095. 25754922 10.1002/sim.6430 PMC4357279

[27] Levin-Rector A (2015) Building-level analyses to prospectively detect influenza outbreaks in long-term care facilities: New York City, 2013–2014. American Journal of Infection Control 43, 839–843. 25960384 10.1016/j.ajic.2015.03.037

[28] Bassett MT and Balter S (2017) Regulating cooling towers to prevent outbreaks of Legionnaires’ disease. Public Health Reports 132, 133–135. 28147210 10.1177/0033354916689612 PMC5349487

[29] Fitzhenry R (2017) Legionnaires’ disease outbreaks and cooling towers, New York City, New York, USA. Emerging Infectious Disease 23. 10.3201/eid2311.161584 PMC5652439 29049017

[30] Peterson ER and Greene SK (2017) Spatio-temporal cluster detection for legionellosis using multiple patient addresses. Online Journal of Public Health Informatics 9, e121.

[31] Greene SK (2015) Disparities in reportable communicable disease incidence by census tract-level poverty, New York City, 2006–2013. American Journal of Public Health 105, e27–e34. 10.2105/AJPH.2015.302741 PMC4539797 26180961

[32] Gleason JA (2017) Analysis of population-level determinants of legionellosis: spatial and geovisual methods for enhancing classification of high-risk areas. International Journal of Health Geographics 16, 45. 29197383 10.1186/s12942-017-0118-4 PMC5712152

[33] Farnham A (2014) Legionnaires’ disease incidence and risk factors, New York, New York, USA, 2002–2011. Emerging Infectious Disease 20, 1795–1802. 10.3201/eid2011.131872 PMC4214295 25513657

